# Wearable smartwatch technology to monitor symptoms in advanced illness

**DOI:** 10.1136/bmjspcare-2017-001445

**Published:** 2017-11-03

**Authors:** Amara Callistus Nwosu, Christopher Quinn, Jamie Samuels, Stephen Mason, Terry R Payne

**Affiliations:** 1 Palliative Care Institute, University of Liverpool, Liverpool, UK; 2 Barrow Hall College, Warrington, UK; 3 St. Margaret’s Church of England Academy, Liverpool, UK; 4 Department of Computer Science, University of Liverpool, Liverpool, UK

**Keywords:** pain management, digital health, information technology, big data, internet of things, personalised health

The development of sensor-based technologies has led to the creation of wearable health monitoring systems to provide real-time feedback information to support health.^1^ This technology has the potential to assist the monitoring and management of symptoms in advanced disease, such as pain management.^2^

Within our university, we have developed a partnership between Computer Science and Palliative Medicine to examine the potential to use technology to support care for people living with advanced illness. One outcome of this collaboration is the development of a prototype smartwatch app to monitor pain experienced by patients. Two school students, completing a summer placement at the University, programmed this software. The app facilitates pain assessment (in numerical rating and descriptive scale formats) and syncs the data with a mobile device to enable storage and retrieval of clinically relevant health data over time.

The King’s Fund has highlighted the potential of new emerging technologies to personalise care to patients according to individual circumstances.^3 4^ For patients living with advanced illness, there is potential to use collected sensor data (for variables such as movement, heart rate and activities of daily living) from wearable devices and living environments, to determine how symptoms (such as pain) affect function and quality of life.^5^

Undoubtedly, the future of healthcare is one in which wearable devices and associated applications will assist the management of health and disease. The challenge for clinicians and computer scientists is to forge collaborative partnerships to understand the needs and opportunities available, and develop acceptable and effective technology.
